# Heterogeneous bone marrow uptake on interim ^18^F-fluorodeoxyglucose positron emission tomography for lymphoma mimicking disease progression: a case report

**DOI:** 10.1186/1752-1947-8-362

**Published:** 2014-11-07

**Authors:** Martin H Cherk, Sushrut Patil, Paul Beech, Victor Kalff

**Affiliations:** 1 Department of Nuclear Medicine, The Alfred Hospital, Commercial Road, Melbourne, Victoria 3004, Australia; 2 Department of Hematology, The Alfred Hospital, Commercial Road, Melbourne, Victoria 3004, Australia; 3 Department of Medicine, Monash University Melbourne, Central Clinical School Alfred Hospital, Commercial Road, Melbourne, Victoria 3004, Australia

**Keywords:** Lymphoma, FDG, PET, Chemotherapy, Response, False, Positive, Granulocyte colony stimulating factor, Pegfilgrastim

## Abstract

**Introduction:**

The use of ^18^F-fluorodeoxyglucose positron emission tomography (^18^F-FDG PET) scanning for baseline staging and assessment of treatment response for higher grade lymphomas is considered to be the standard of care. Evaluation of lymphomatous bone marrow infiltration on ^18^F-FDG PET can usually distinguish between normal regenerating marrow following chemotherapy by a characteristic pattern of uptake.

**Case presentation:**

Here we report the case of a 51-year-old Caucasian woman with mixed low- and high-grade lymphoma with biopsy confirmed marrow infiltration. An interim post-three cycle chemotherapy ^18^F-FDG PET scan revealed apparent progression of marrow disease. Subsequent investigations were performed including bone marrow biopsies, repeat ^18^F-FDG PET scanning and a white cell scan. These revealed the interim ^18^F-FDG PET scan appearance was due to a highly unusual pattern of scattered islands of regenerating normal marrow, rather than progressive lymphoma.

**Conclusions:**

Our case report highlights that apparent severe bone marrow abnormalities on ^18^F-FDG PET scans in lymphoma patients treated with chemotherapy are not always due to disease. Clinicians should retain a high index of suspicion for benign causes when ^18^F-FDG PET scan results appear incongruent with clinical response.

## Introduction

The use of ^18^F-fluorodeoxyglucose positron emission tomography (^18^F-FDG PET) scanning for baseline staging and assessment of treatment response for higher grade lymphomas is considered standard of care [1,2], and in many institutions has replaced diagnostic computed tomography (CT) as the imaging modality of choice for this condition. There is overwhelming evidence in the literature demonstrating the powerful prognostic utility of ^18^F-FDG PET with higher grade lymphoma patients who have a persistently positive interim mid-treatment (post-two or three cycles of chemotherapy) PET scan for disease having a significantly worse prognosis compared to those who achieve complete metabolic remission [3-5]. As a result, many clinical trials are currently being conducted to evaluate whether a change or intensification in therapy, such as stem cell transplantation in patients with a positive interim PET scan, confers survival benefit [6-8].

Although spurious results can occur due to cellular uptake of ^18^F-FDG not being specific to lymphoma or malignancy, a false positive finding on interim PET scanning is generally low and in equivocal cases can often be resolved with tissue sampling and histological correlation.

^18^F-FDG PET scanning is quite sensitive for detecting bone marrow involvement in higher grade lymphomas and is typically associated with a heterogeneous pattern of increased marrow activity within the skeleton [9,10]. In contrast, physiologic reactive marrow, which is often seen following blood loss, sepsis or shortly after chemotherapy, is usually depicted by diffuse relatively uniform increased FDG uptake throughout the axial and proximal appendicular skeleton [11].

## Case presentation

In February 2013, a 51-year-old Caucasian woman presented with a several month history of back pain, hot flushes, sweats and weight loss. A subsequent CT scan demonstrated multiple enlarged cervical nodes, as well as confluent paravertebral soft tissue masses in the region of the thoracic spine and anterior to L5 to the presacral region. A CT-guided core biopsy of the presacral mass was performed and histology confirmed low grade B-cell non-Hodgkin lymphoma based on immunostaining results.

**Figure 1:**
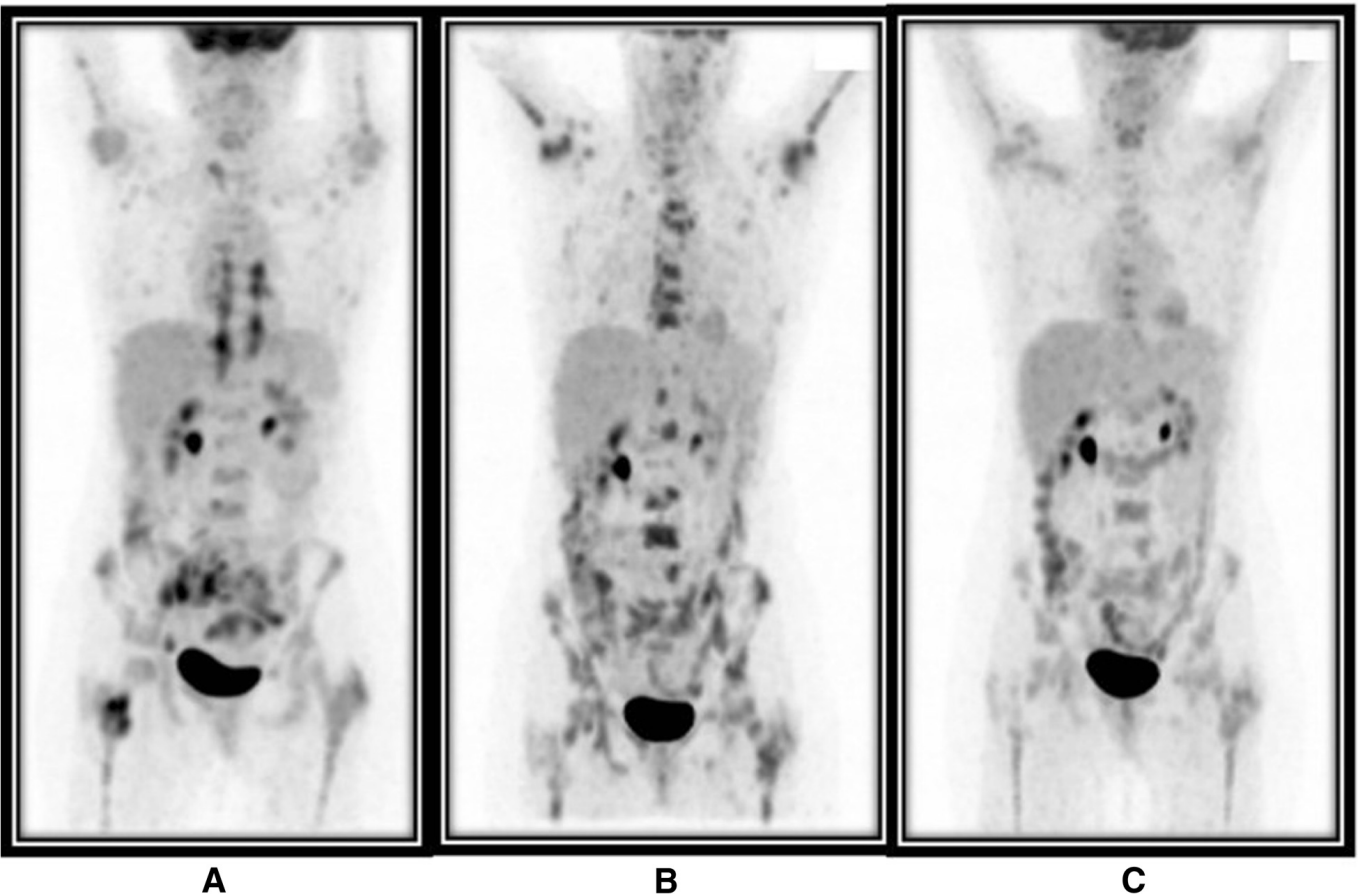
**Three-dimensional maximal intensity projection **^**18**^**F-fluorodexoyglucose positron emission tomography scan images.** Baseline prior to treatment **(A)**, after three cycles of chemotherapy **(B)** and at completion of six cycles of chemotherapy **(C)**.

Further staging included a whole body ^18^F-FDG PET scan which demonstrated moderate to markedly FDG-avid right upper and bilateral lower thoracic para-spinal, lower lumbar para-spinal and presacral soft tissue masses with extension into several right lower lumbar and sacral neural foramen. Heterogeneous increased FDG uptake was also seen in a right external iliac node and throughout the skeleton, most marked and intense in the trochanteric region of the right femur and right ilium, consistent with marrow infiltration (Figure 1A). A bone marrow aspirate and trephine of the right ilium at a site of increased FDG uptake on the PET scan confirmed a marrow packed with lymphoma, with a combination of small and larger cells, the latter of which were CD20 positive. Due to sacral and lumbar neural foramen invasion, CT and magnetic resonance imaging (MRI) brain scans along with a lumbar puncture were performed to exclude leptomeningeal disease. These were found to be negative.

Despite the presacral mass core biopsy demonstrating low grade B-cell small lymphocytic lymphoma, clinical symptoms, bone marrow biopsy and ^18^F-FDG PET scan findings were suggestive of concomitant higher grade lymphoma. She was thus considered likely to have follicular B-cell lymphoma with high-grade transformation and treatment comprised of six cycles of cyclophosphamide, doxorubicin, vincristine, prednisolone and rituximab (CHOP-R) chemotherapy with pegfilgrastim support every three weeks. This was followed by two cycles of high-dose methotrexate due to the perceived higher risk for central nervous system disease from direct neural foramen invasion.

**Figure 2:**
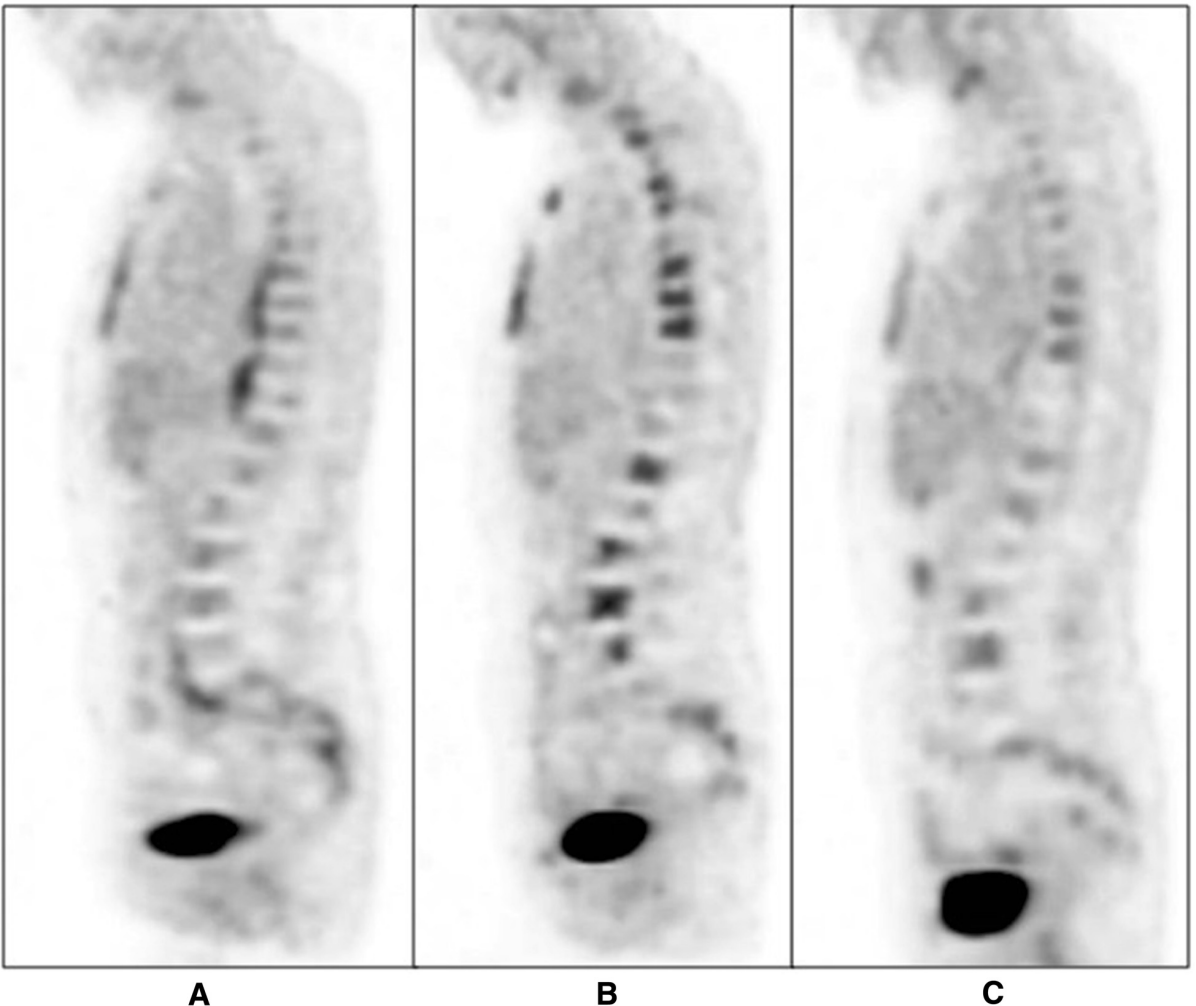
**Midline sagittal positron emission tomography scan images highlighting pattern of **^**18**^**F-fluorodexoyglucose uptake in bone marrow of the spine.** Baseline prior to treatment **(A)**, after three cycles of chemotherapy **(B)** and at completion of six cycles of chemotherapy **(C)**.

The first three cycles of CHOP-R chemotherapy were well-tolerated without significant complication apart from persistent low-grade lower back pain. An interim mid-treatment ^18^F-FDG PET scan to assess treatment response was performed at this point which demonstrated resolution of FDG-avid right upper and bilateral lower thoracic para-spinal, lower lumbar para-spinal and pre-sacral soft tissue masses and right external iliac node (Figure 1B). FDG uptake in the trochanteric region of the right femur and right ilium had also decreased, however, new heterogeneous intense FDG uptake was demonstrated throughout most of the axial and proximal appendicular skeleton, sternum and numerous ribs. Some of this corresponded with mixed lytic and sclerotic lesions on the fusion low-dose CT scan performed at the time of the ^18^F-FDG PET scan which was highly suggestive of widespread high-grade lymphoma progression within the marrow (Figures 1B and 2B).

A right posterior superior iliac spine bone marrow biopsy was thus performed with a view to intensifying chemotherapy if persistent lymphoma was confirmed within the marrow. This was performed at a site previously positive for marrow involvement on the pretreatment PET scan, and demonstrated a moderately hypocellular marrow with some clusters of B-cells, which may have been regenerative in nature. No convincing evidence of residual lymphoma was seen. Given the suspicious PET scan findings and potential patchy nature of disease, a repeat bone marrow biopsy was performed from the left posterior superior iliac spine, which again demonstrated no evidence of lymphoma and normal moderately hypercellular tri-lineage hemopoiesis.

**Figure 3:**
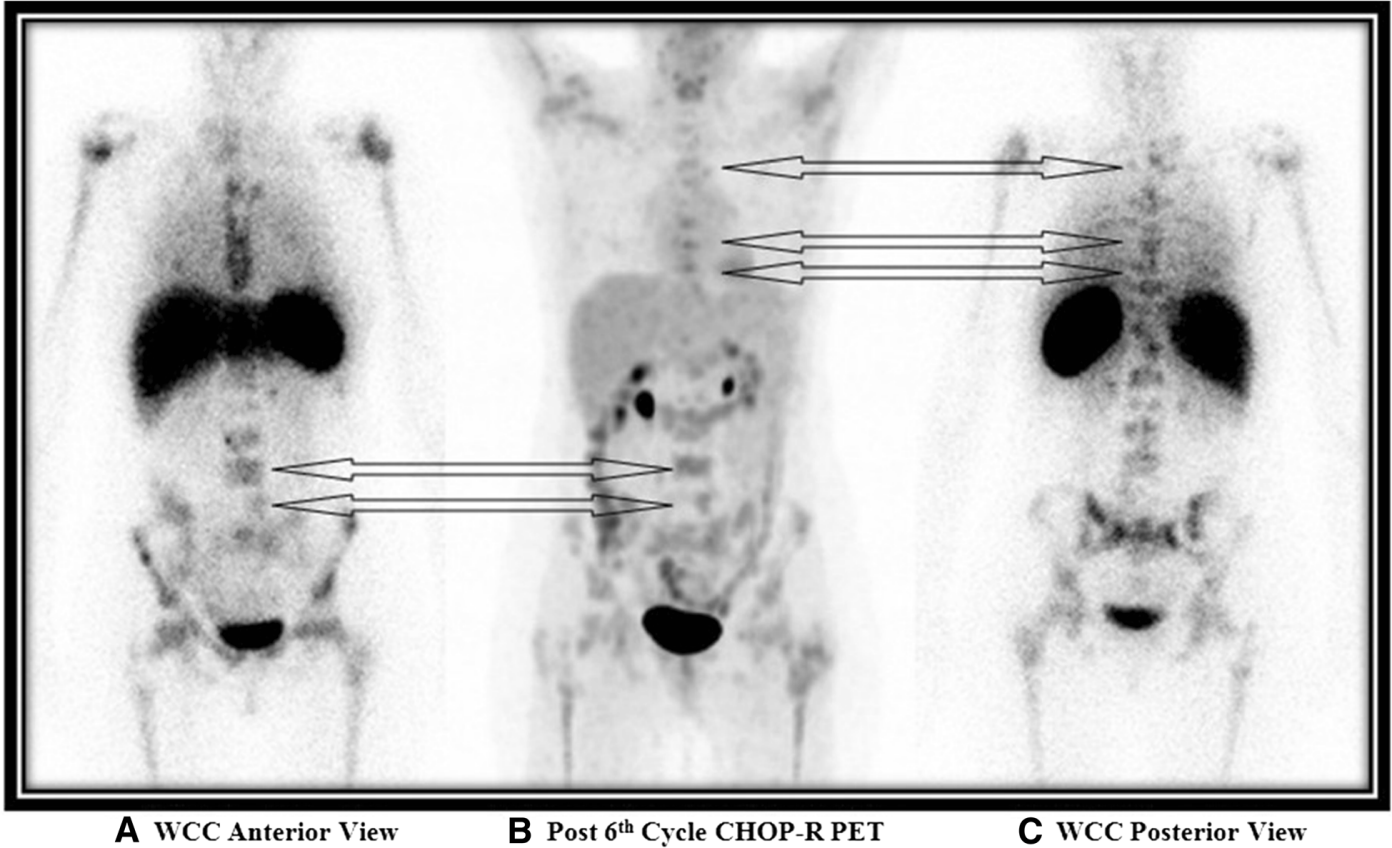
**Comparison of **^**18**^**F-fluorodexoyglucose positron emission tomography scan uptake in spine with sites of normal bone marrow activity on whole body white cell scan post six cycles of chemotherapy.** White cell scan anterior planar view **(A)**, White cell scan posterior planar view **(C)** three-dimensional maximal intensity projection (MIP) ^18^F-FDG PET scan images **(B)**. The overall pattern of FDG uptake on PET scan is similar to the white cell scan uptake in keeping with sites of normal regenerating marrow rather than lymphoma. Arrows highlight similarities in FDG and white cell uptake in the thoracic and lumbar spine.

As residual lymphoma was not confirmed on both bone marrow biopsies, treatment was not changed and she completed a further three cycles of CHOP-R chemotherapy, at which point another ^18^F-FDG PET scan was performed (Figures 1C and 2C). This scan again demonstrated a similar heterogeneous pattern of increased FDG uptake throughout the marrow of the skeleton in a similar distribution to the post third cycle interim PET scan however, overall intensity of uptake had decreased significantly and was not typical for disease progression.Due to the apparent improvement in PET scan appearances, a whole body radiolabelled white cell scan was performed to evaluate normal skeletal bone marrow activity. This demonstrated areas of normal marrow activity throughout the skeleton in a similar highly heterogeneous distribution to the interim and post-six cycle PET/CT scans. This suggested that the apparent ‘abnormal’ bone marrow appearance on the interim and, to a lesser extent, post-six cycle chemotherapy PET scans actually represented FDG uptake in scattered residual islands of regenerating and pegfilgrastim-stimulated normal bone marrow, rather than sites of progressive lymphoma (Figure 3).

Our patient has remained in clinical remission for three months since the post-six cycle chemotherapy PET scan, and continues to receive three-monthly rituximab maintenance therapy for the presumed lower grade lymphoma component of her disease.

The baseline, interim and completion PET scans were all performed on the same Phillips GEMINI PET/CT camera (Phillips Healthcare, Massachusetts, United States). The range of ^18^F-FDG dose and uptake time for the three PET/CT scans were 307 to 319 MBq and 60 to 65 minutes, respectively, suggesting differences between PET scan appearances were true changes and not due to variability in the acquisition technique.

## Discussion

This case highlights assessment of treatment response in bone marrow on PET scanning can be confounded in cases where the lymphoma has caused significant disruption to normal marrow architecture, resulting in islands of normal residual rapidly regenerating marrow giving a ‘pathological’ appearance. In this circumstance a further bone marrow scan, in this case a white cell scan, is useful in discriminating between bone marrow regeneration and progressive disease by the degree of its congruence with the FDG PET study.

Our case is unusual as the focal increased marrow and bony FDG uptake in the trochanteric region of the right femur and right ilium was consistent with disease involvement on the baseline pretreatment PET scan. FDG uptake in the remainder of the skeleton appeared relatively normal despite mixed lytic and sclerotic lesions scattered throughout the skeleton on CT. The lack of FDG-avidity at these sites suggested an underlying lower grade lymphoma, with the more FDG-avid sites representing sites of transformed higher grade disease. The interim post-three cycle chemotherapy PET scan was most concerning for a mixed response to therapy, with the new highly heterogeneous appearance in the marrow being suspicious for disease progression despite metabolic resolution of the femoral and ilial bone lesions and sites of nodal disease.

The typical normal reactive marrow appearance seen following chemotherapy is that of diffusely increased relatively uniform FDG uptake throughout the marrow [11]. In patients with malignant marrow infiltration or bone involvement, successfully treated areas usually appear photopenic or inactive on follow-up PET scans. This can result in a heterogeneous appearance where sites of successfully treated lymphoma which appear photopenic are interspersed with islands of more FDG-avid regenerating normal marrow. This ‘flip-flop’ phenomenon on PET scan has been previously described in cases of high-grade lymphoma [12,13]. In most cases of lymphomatous involvement of bone or bone marrow, the heterogeneous appearance on post-therapy PET scans can usually be reconciled from being malignant in nature by carefully comparing pre- and post-treatment PET scan appearances [14].

As our patient likely has mixed low- and high-grade lymphoma, the interim and post-therapy PET/CT scan appearances possibly represent a partial flip-flop variant, with photopenic sites representing either sites of successfully treated disease or non FDG-avid less chemotherapy responsive lower grade lymphoma.

A further likely contributing factor in this case is the timing of PET scanning post-pegfilgrastim administration. The post-three cycle interim PET scan was performed four days following pegfilgrastim administration. At this time point, pegfilgrastim was likely having its maximal stimulatory effect on granulopoiesis, resulting in any residual islands of normal bone marrow appearing particularly FDG-avid, further mimicking disease progression in the marrow [15,16]. In contrast, the post-six cycle chemotherapy PET scan was performed 17 days following pegfilgrastim administration, a time point where pegfilgrastim effects would have subsided resulting in any residual islands of normal bone marrow appearing less FDG-avid. The findings of this post-six cycle PET study showed strong concordance with the subsequently performed white cell scan, with the distribution and intensity marrow lesions now being essentially equivalent.

## Conclusions

Apparent new focal bone marrow abnormalities on PET scans in lymphoma patients treated with chemotherapy and pegfilgrastim are not always due to progressive disease. Clinicians should retain a high index of suspicion for benign causes, particularly if PET results show metabolic remission of soft tissue and/or nodal disease. In such cases, an additional bone marrow scan, such as a white cell scan or a Tc-99m sulphur colloid scan [17], may be useful in differentiating benign regenerating marrow from true disease progression.

## Consent

Written informed consent was obtained from the patient for publication of this Case report and any accompanying images. A copy of the written consent is available for review by the Editor-in-Chief of this journal.

## Abbreviations

^18^F-FDG PET: ^18^F-fluorodeoxyglucose positron emission tomography; CT: Computed Tomography; Tc-99m WCC: Technetium-99m labelled white cell scan.

## Competing interests

The authors declare that they have no competing interests.

## Authors’ contributions

MC was involved in the analysis and interpretation of medical imaging and drafting the manuscript. SP was involved in treating the patient and revising the manuscript. PB and VK were involved in performing, analysis and interpretation of medical imaging and revising the manuscript. All authors read and approved the final manuscript.
